# Shining in the dark: First record of biofluorescence in the seahorse *Hippocampus reidi*

**DOI:** 10.1371/journal.pone.0220561

**Published:** 2019-08-08

**Authors:** Amanda C. Vaccani, Natalie V. Freret-Meurer, Áthila A. Bertoncini, Luciano N. Santos

**Affiliations:** 1 Departamento de Ecologia e Recursos Marinhos, Universidade Federal do Estado do Rio de Janeiro, Rio de Janeiro, Rio de Janeiro, Brazil; 2 Instituto de Biologia, Universidade Santa Úrsula, Rio de Janeiro, Rio de Janeiro, Brazil; Universidade Federal de Uberlândia, BRAZIL

## Abstract

Marine environments are visual domains restricted regarding light characteristics. Overall, blue monochromatic spectrum prevails in offshore areas especially below 15m depth, since long wavelengths are quickly attenuated. Light intensity is even more constrained in coastal waters, particularly those of tropical estuaries and bays, because further scattering through dissolved and suspended materials. Biofluorescence, which is the ability of organisms to absorb light and reflect it in a different wavelength, has been reported for many marine fish. In this paper, biofluorescence was recorded for the first time for the longsnout seahorse *Hippocampus reidi*, under natural conditions at Ilha Grande bay, Brazil, and both adult, juvenile and fry individuals kept in captivity. Although displaying the same colour emissions, seahorses differed in relation to body lighting, colour patterns, and age wherein fluorescence occurs. Newborn seahorses exhibit green biofluorescence only in the eyes and stomach. Further experiments are necessary to address whether *H*. *reidi* can change the patterns of biofluorescence emission for sensorial and social purposes.

## Introduction

Marine environment has visually restricted domain when compared to terrestrial environments. Overall, blue monochromatic spectrum prevails in offshore areas, especially below 15m depth, since long wavelengths within the red-orange range are quickly attenuated by saltwater in shallower layers. Light intensity is even more constrained in coastal waters, particularly those of tropical estuaries and bays, because further scattering through dissolved and suspended materials [1]. Consequently, many species living in these systems display particular mechanisms, such as biofluorescence, which might contribute for sensorial and social functions during lighting restrictive conditions [2].

Biofluorescence is the absorption of electromagnetic radiation at a specific wavelength, followed by their resubmission at a longer or shorter wavelength [3]. Biofluorescence occurrence has been largely reported, both for terrestrial and marine animals, but there is still little information about its functionality, although it is probably related to intraspecific communication [4,5] and camouflage [3]. This phenomenon has been reported for several marine organisms, especially invertebrates and fish [5,6,7,8], but its occurrence for tropical marine fish at natural conditions remains barely known.

Among reef fishes, seahorses (Syngnathidae family), are widespread in tropical and subtropical waters, and 42 species are currently known [9,10]. Three species are recorded in Brazil, the longsnout seahorse *Hippocampus reidi* Ginsburg, 1933, the shortsnout seahorse *Hippocampus erectus* Perry, 1810, and the most recently described Patagonian seahorse *Hippocampus patagonicus* Piacentino and Luzzatto, 2004. Although *H*. *reidi* is the most common seahorse species in Brazil, it has been regarded as *vulnerable* by the List of Threatened Species of the Brazilian Ministry of the Environment [11] and as *near threatened* by the Red List of the International Union for Conservation of Nature [12].

Worldwide, biofluorescence was recently reported for the Syngnathidae family, and only to four seahorse species, *H*. *erectus*, *Hippocampus zosterae* Jordan and Gilbert, 1882 [3], *Hippocampus bargibanti* Whitley, 1970 and *Hippocampus denise* Lourie and Randall, 2003 [13]. The present study aimed to check for biofluorescence in the seahorse *H*. *reidi* in both natural and captivity, describing its colour and body location. Potential implications on sensorial and social functions are briefly discussed.

## Materials and methods

**Fig 1:**
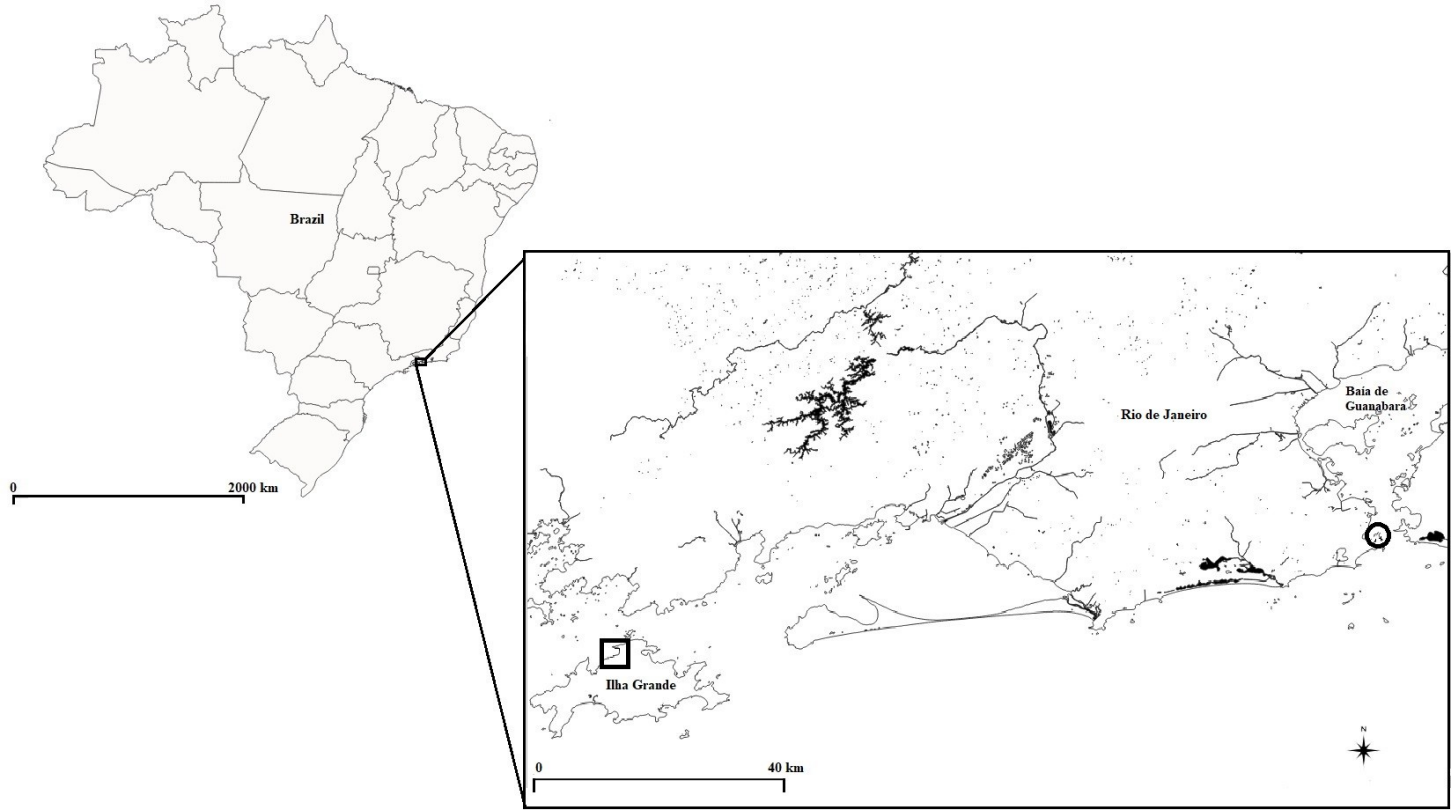
Place where animals were collected and where animals were observed in the wild. Locations where seahorses were collected (circle) and observed in the wild (square).

Experiments in captivity were conducted with *H*. *reidi* captured in natural environment. Five *H*. *reidi* were caught (SISBio license code 56695–1) in February 2018 by a snorkeling diver on the rocky shores of Urca beach (22° 56'33 "S—043° 09'27" W), Guanabara bay, Rio de Janeiro, Brazil (Fig 1). All animals (*n =* 6) used in experiments were returned to the same natural catch area, and there was no death of any individual. They were stored alive in plastic bags with salt water and carried to the Laboratory of Animal Behavior and Conservation, at Santa Ursula University, Rio de Janeiro. They were acclimatized for three days in aquarium at constant water conditions (Table 1). Twenty-for hours before each experiment, the animals were cleaned manually, through softly friction with fingers, and then macroscopically checked to exclude any sign of epiphytes in the animal’s body.

**Table 1:** Environmental variables measured during experiments and observations.

Variables	Laboratory	Field
Temperature (°C)	28.0	29.2
Salinity	33.0	33.8
pH	8.1	9.0
Ammonium (ppm)	0	Not measured
Nitrite (ppm)	0	Not measured
Nitrate (ppm)	0	Not measured

Environmental variables measured during exposure of seahorses to 450 nm wavelength in laboratory and field trials.

This study was approved by the Ethics Committee on the use of Animals (CEUAUSU00004) and followed the ‘Ethical Principles in Animal Research’ guidelines adopted by the National Council of Animal Experimentation Control (CONCEA).

Experiments were conducted with two females, one male *H*. *reidi*, and two juveniles stocked in 80L-aquarium filled with saltwater. During the experiments seahorses were kept alone in the aquarium in complete darkness, lightened only by the Night Sea excitation (royal blue exposure: ~450 nm), with a yellow barrier filter that completely blocks the reflected excitation of 440–460nm wavelengths. The biofluorescence was recorded by a Canon EOS 5D mark IV camera and a Canon EF 100mm ƒ/2.8LIS USM Macro lens.

A second experiment was performed using another pregnant male captured in September 2018 in Urca beach, Guanabara bay. The adult *H*. *reidi* was inspected for the occurrence of biofluorescence, and then the newborn seahorses were assessed during two days from their birth. The same aquarium conditions and equipment were used in this experiment, which occurred in October 2018.

Biofluorescence was registered in the field by two scuba divers between 6:00 and 9:00 pm on the rocky shores of Bananal beach (23°06’28.24” S e 44°14’57.16” W), Ilha Grande bay, in March 2018 (Fig 1). Three (two female and one male) individuals found in field were photographed with the same equipment used in laboratory.

The biofluorescence was qualified through the images analysis according to the body parts (head, trunk and tail) and colors displayed. We quantified fluorescence using the PhotoQuad software, where 100 points are randomly cast in each part of the body and classified according to the displayed color.

## Results

**Fig 2:**
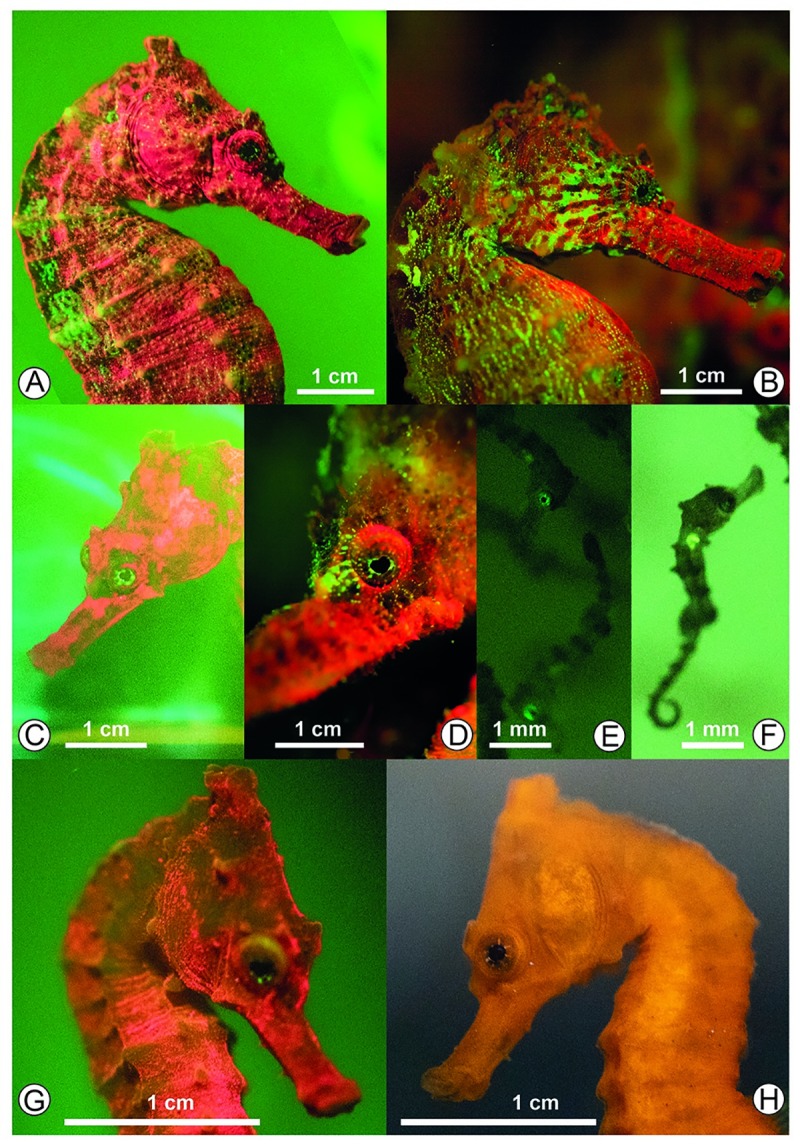
Seahorses in laboratory and field displaying biofluorescence. *Hippocampus reidi* exhibiting biofluorescence in green and red spectrum in laboratory (A) and field (B) conditions. Male seahorses of 101 mm (A) shows dots of green biofluorescence in the trunk and head areas, while male seahorse (B) 123 mm, shows stripes of green biofluorescence in the trunk and head areas. Biofluorescence lighting in green spectrum in the eyes in laboratory (C) and field (D) conditions. Two-day-old seahorses showing no biofluorescence (E,F). A young seahorse, 4.3cm, exhibits mostly red biofluorescence under exciting light (G). The same individual is depicted in orange under natural white light (H). Photos: AAB.

In laboratory, all adults and juveniles (*n* = 6) showed red and green biofluorescence. Light emissions were observed over the trunk, on both eyes, sides and tail (Fig 2A and 2C). Green biofluorescence was more evident on the eyes and as small dots all over the body, while red color was found covering all over the head, trunk and tail. This pattern was the same for all but intensity varied among them. Pregnant male also showed the same pattern of biofluorescence found for the other seahorses, both in relation to color emission as for body location of lighting. Some individuals had small green dots on the trunk and tail, but it was not a pattern exhibited by all observed individuals.

**Fig 3:**
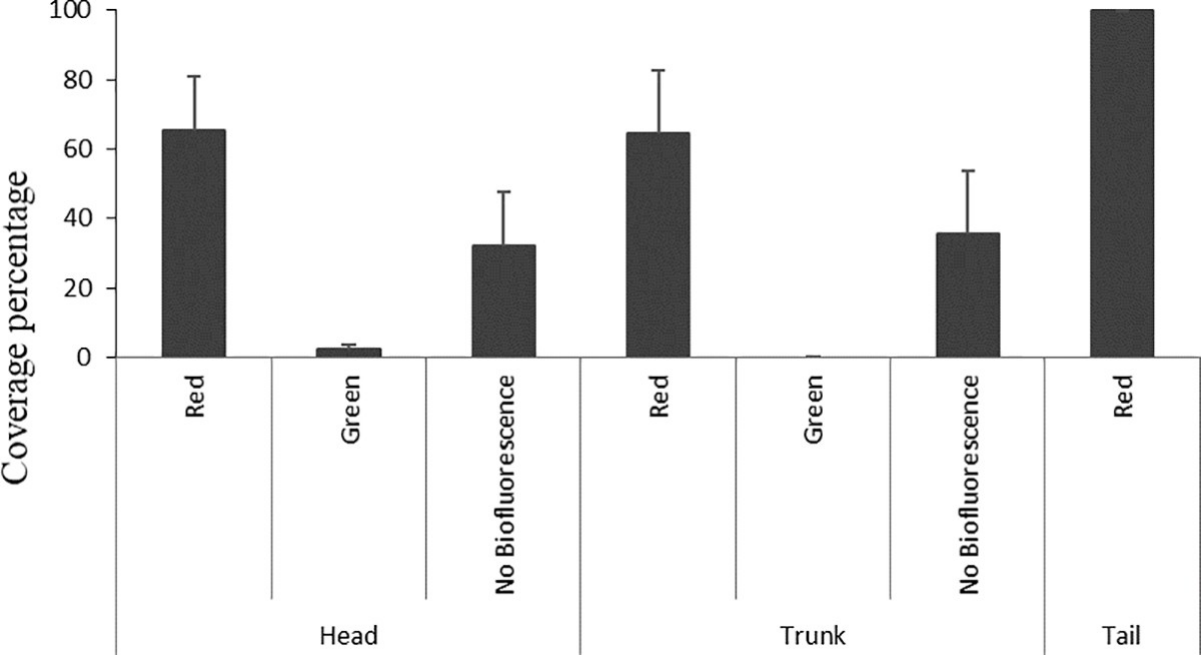
Color cover on adult’s body. Percentage of color cover on body parts in adult individuals in laboratory.

Adult individuals had a higher percentage of red fluorescence both on the head (65.5%) and on the trunk (64.5%). The green color was only displayed on the head (2.3%) and the tail was 100% red (Fig 3). Other parts on the head and trunk presented no biofluorescence with 32.3% and 35.5% cover, respectively.

The biofluorescence was not quantified for the individuals observed in the field, because they grasped on the holdfast and hid part of their body becoming impossible to analyze it in the images, therefore we only qualified according to the body part and coloration displayed.

**Fig 4:**
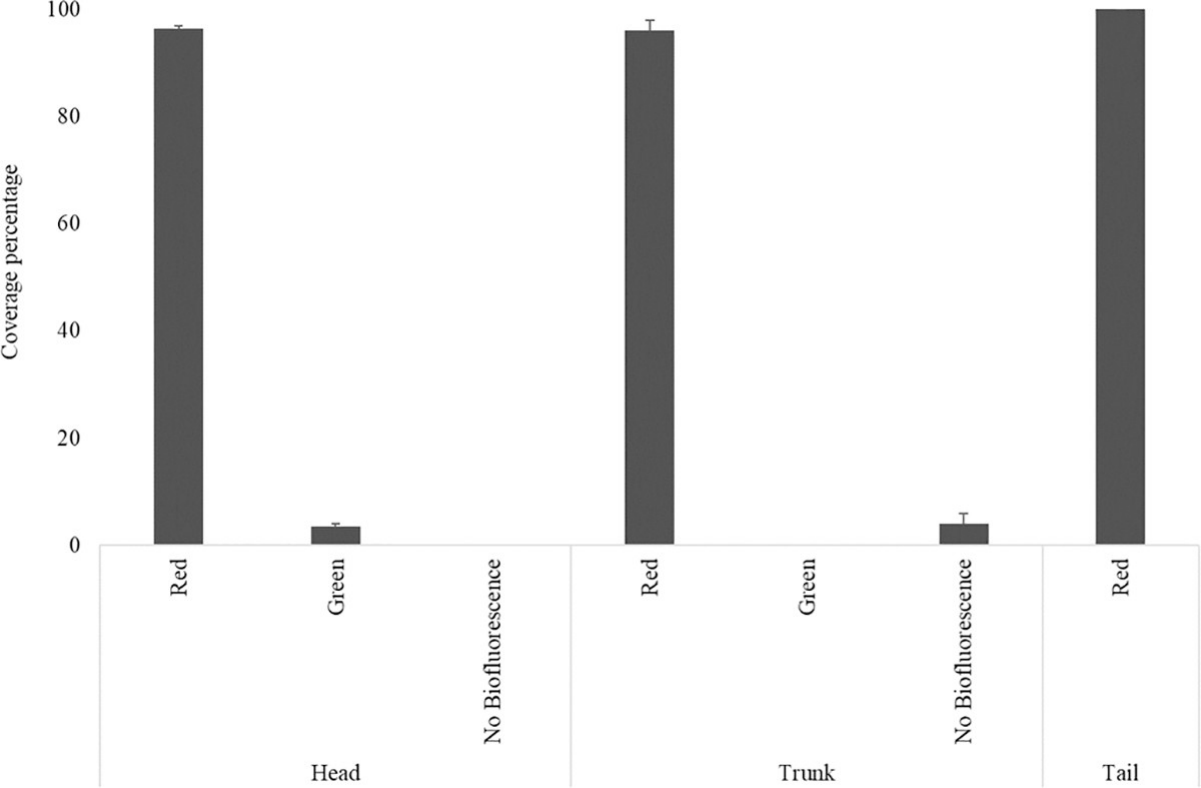
Color cover on juvenile’s body. Percentage of color cover on body parts in juveniles individuals in laboratory.

Two young individuals were analyzed, which presented the major part of the body with red biofluorescence (head– 96,5%; trunk– 96%; tail– 100%) (Fig 4). Green fluorescence has only been observed on the eyes.

Biofluorescence was recorded for all the 1.830 newborn seahorses, with green biofluorescence on the eyes and the stomach (Fig 2G). Our results indicate that biofluorescence already is present in the first two days of life in *H*. *reidi* (Fig 2E and 2F).

In the field, three individuals were observed presenting green and red fluorescence as displayed in the laboratory. Green biofluorescence was observed on the eyes, also in the way of stripes along the head and on lateral side of the body of some individuals (Fig 2B). Red biofluorescence was observed throughout the whole *H*. *reidi* body (Figs 2B and 1D), however it changed according to the green biofluorescence exhibition. Water characteristics in field were similar to the conditions maintained in the aquariums (Table 1).

## Discussion

Our study was the first to report biofluorescence for the longsnout seahorse *H*. *reidi* both in nature and captivity. Although displaying the same colour emissions (green and red), seahorses differed in relation to body lighting, colour patterns, and age at which fluorescence occurs. In laboratory, seahorses showed green biofluorescence in head and some individuals showed dots in the trunk, contrasting with seahorses in nature, which showed stripes of green biofluorescence in the trunk and head areas.

Our findings agree with those presented for other four seahorse species (*H*. *erectus*, *H*. *zosterae*, *H*. *bargibant* and *H*. *denise*) and also pipefish species of the Syngnathidae family, in relation to the occurrence of green and red biofluorescence [13,14,7,3].

Red fluorescence was recorded covering the head, trunk and tail of seahorses in our study. Red fluorescence, ~600 nm of light spectrum, was detected for several marine species [14], but it has been recorded only for a few numbers of fish species [7,15,16,17,3,18]. This pattern disagrees partially to Michiels et al.[7], who described red biofluorescence more associated with the head and eyes of several reef fish species, whereas the eyes of *H*. *reidi* were always lighting with green fluorescence. Michiels et al.[7] suggest that biofluorescence, especially the red spectrum, is related to intraspecific communication. Red fluorescence encompasses a wavelength range largely unavailable within the marine environment, being quickly attenuated with depth, especially in turbid systems, such as tropical bays and estuaries. Seahorses are visual animals, which use coloring in different context, such as courtship and camouflage [19].

According to Sparks et al.[3] in addition to red fluorescence, previously reported in fish associated with shallow reefs, marine fish also exhibit combinations of green, orange and red fluorescence, and some only green coloring, in unique patterns and species specific.

Green biofluorescence was recorded in a lesser extent for seahorses of both natural and captivity, occurring in the eyes of all individuals and as stripes and small dots spread in the trunk. Sparks et al.[3] reported green fluorescence covering the entire body of fishes of other families, contrasting with the patterns found for seahorses in the present study and for *H*. *bargibant* and *H*. *denise* [13], especially in relation to the eyes. Green fluorescence has been reported to attract preys in hydromedusa and has an effective result during day light [20]. Considering that *H*. *reidi* has green fluorescence mainly concentrated on the head, eyes and upper trunk, and it is a diurnal species, it is possible that green fluorescence is related to prey attraction. Notably, younger individuals showed a very simple color pattern under fluorescence and under natural light conditions.

Our findings suggest thus that *H*. *reidi* might use biofluorescence as a complementary tool for communication, such as to contrast itself with the sea bottom and interact with other individuals at short distances. However, further studies should be performed to investigate whether this ability is important or not, since biofluorescence has already been detected for newborn seahorses of two-days age.
